# Analysis of Bacteriophage Behavior of a Human RNA Virus, SARS-CoV-2, through the Integrated Approach of Immunofluorescence Microscopy, Proteomics and D-Amino Acid Quantification

**DOI:** 10.3390/ijms24043929

**Published:** 2023-02-15

**Authors:** Carlo Brogna, Vincenzo Costanzo, Barbara Brogna, Domenico Rocco Bisaccia, Giancarlo Brogna, Marino Giuliano, Luigi Montano, Valentina Viduto, Simone Cristoni, Mark Fabrowski, Marina Piscopo

**Affiliations:** 1 Department of Research, Craniomed Group Facility Srl., 20091 Bresso, Italy; 2 Biogem, Institute of Molecular Biology and Genetics, 83031 Ariano Irpino, Italy; 3 Department of Radiology, Moscati Hospital, Contrada Amoretta, 83100 Avellino, Italy; 4 Marsanconsulting Srl. Public Health Company, Via dei Fiorentini, 80133 Napoli, Italy; 5 Andrology Unit and Service of LifeStyle Medicine in Uro-Andrology, Local Health Authority (ASL), 84124 Salerno, Italy; 6 Long COVID-19 Foundation, Brookfield Court, Garforth, Leeds LS25 1NB, UK; 7 ISB—Ion Source & Biotechnologies Srl., 20091 Bresso, Italy; 8 Department of Emergency Medicine, Royal Sussex County Hospital, University Hospitals Sussex, Eastern Road, Brighton BN2 5BE, UK; 9 Department of Biology, University of Naples Federico II, 80126 Napoli, Italy

**Keywords:** SARS-CoV-2, microbiome, feces, fluorescent microscopy, spectral counting, D-Amino acid, mass spectrometry, nasopharyngeal swab, bacteriophage

## Abstract

SARS-CoV-2, one of the human RNA viruses, is widely studied around the world. Significant efforts have been made to understand its molecular mechanisms of action and how it interacts with epithelial cells and the human microbiome since it has also been observed in gut microbiome bacteria. Many studies emphasize the importance of surface immunity and also that the mucosal system is critical in the interaction of the pathogen with the cells of the oral, nasal, pharyngeal, and intestinal epithelium. Recent studies have shown how bacteria in the human gut microbiome produce toxins capable of altering the classical mechanisms of interaction of viruses with surface cells. This paper presents a simple approach to highlight the initial behavior of a novel pathogen, SARS-CoV-2, on the human microbiome. The immunofluorescence microscopy technique can be combined with spectral counting performed at mass spectrometry of viral peptides in bacterial cultures, along with identification of the presence of D-amino acids within viral peptides in bacterial cultures and in patients’ blood. This approach makes it possible to establish the possible expression or increase of viral RNA viruses in general and SARS-CoV-2, as discussed in this study, and to determine whether or not the microbiome is involved in the pathogenetic mechanisms of the viruses. This novel combined approach can provide information more rapidly, avoiding the biases of virological diagnosis and identifying whether a virus can interact with, bind to, and infect bacteria and epithelial cells. Understanding whether some viruses have bacteriophagic behavior allows vaccine therapies to be focused either toward certain toxins produced by bacteria in the microbiome or toward finding inert or symbiotic viral mutations with the human microbiome. This new knowledge opens a scenario on a possible future vaccine: the probiotics vaccine, engineered with the right resistance to viruses that attach to both the epithelium human surface and gut microbiome bacteria.

## 1. Introduction

It has been three years since the pandemic was proclaimed to be caused by the SARS-CoV-2 coronavirus. Many scientists have shown countless methods of searching for the viral RNA of this coronavirus [1,2,3,4]. Many of these approaches are molecular tests, and “the gold standard” is considered the real-time qRT-PCR (Real-Time Quantitative Reverse Transcription PCR) [4]. Some authors have emphasized the importance of collecting biological samples from the nasopharyngeal tract [5,6,7]; others have recently observed very little difference in sensitivity and specificity for molecular tests on nasopharyngeal biological samples and those on salivary samples [8,9,10]. One of the disadvantages of molecular testing is that it probably requires continuous updating of the genetic probes which recognize the viral genome because of the continuous mutations described during virus sequencing. It should be considered that continuous mutations in the target regions of primers and probes induced by continuous virus mutations can affect the accuracy of molecular tests [11].

In the past, a rapid method to find coronaviruses in feces was the microscopic technique of the hemadsorption-eluting-hemagglutination (HEHA) test [12]. It consisted of using antibodies in serum to specifically agglutinate viral particles to be observed agglomerated using an electron microscope. This method was counterbalanced by the fluorescent viral precipitin assay [13,14], a simpler technique used by laboratories not equipped with electron microscopes. This approach allowed the detection of virus–antibody aggregates and did not require an electron microscope but required serum from animals already immunized against the virus being studied.

Nowadays, fluorescence and electron microscopy represent two useful and reliable approaches for detecting and searching viral particles in biological fluids. In particular, fluorescence microscopy is a milestone in the field of microscopy. This has been instrumental in elucidating cellular pathophysiology both in vitro [15,16,17] and in vivo [18,19,20], as well as in testing the efficacy of pharmacological compounds for researching genetic disorders [21,22]. Fluorescence microscopy was used to show images of the organs of a 14-month-old child who died from SARS-CoV-2 [23]. Later, immunofluorescence showed the kinetics of virus replication in Vero E6-Cells [24]. The authors in [25] emphasize the importance of immunofluorescence and its use in the study of SARS-CoV-2 replication and infection patterns. At the same time, the authors in [26] were among the first to show, by confocal microscopy, images of the SARS-CoV-2 virus in the nasal epithelium.

New observations on SARS-CoV-2 and its RNA increase phenomenon have been described, including in 30-day-old fecal bacterial cultures and prokaryotic cells [27,28]. The authors showed the contamination through the oral–fecal route through in vitro experiments and the increase of RNA viral load in bacterial cultures for up to 30 days of testing. From this evidence, there appears to be a strong correlation between bacteria in the human gut microbiome and SARS-CoV-2. Many laboratories are not equipped with electron microscopy, while fluorescence microscopy is used in research centers. These methods require experienced staff and high costs.

Classically, there are several methods for quantifying bacteriophages: measuring virus infectivity, measuring nucleic acids or proteins, or physically counting particles [29].

During the study of bacteriophage viruses in general, genetic quantification of the virion may not coincide with the real abundance of the pathogen in the sample under analysis. It should be considered that in case of the lysogenic or moderate appearance of the virus, its genome will be present, but its protein expression will not cease. Mass spectrometry makes it possible to overcome this obstacle. In other words, it verifies whether the protein is formed and, thus, whether the virion is present in its final form—allowing one to approach the second step: immunofluorescence microscopy or electron microscopy.

Combining today’s more versatile methods can help to better understand bacteriophage phenomena in the bacteria derived from the human microbiome, which are often underestimated. This study underlines the application of mass spectrometry in the search for viral proteins and quantification by means of a well-established procedure—spectral counting [30] and immunofluorescence microscopy. The problem of cross-reactivity of antibodies used in immunofluorescence microscopy, in addition to being routinely solved by control tests, can be eliminated by searching for the proteins by mass spectrometry. For more than 80 years, mass spectrometry has been known as one of the most sensitive methods in the literature. Although a genetic probe can determine the presence of the virus RNA in general, in a biological sample, mass spectrometry allows verification of the presence of the protein produced, and immunofluorescence can then highlight the iconographic aspect of the interactions. Spectral counting is a semi-quantitative evaluation with respect to conditions of absent or low protein and/or peptide presence [31], and it is possible to assess in vitro bacterial cultures, increased or decreased protein concentration under conditions of infection and growth of the peptide numbers of viral particles. Compared with statistical analysis and immunofluorescence microscopy in samples, this method allows the data to be cross-referenced, and each represents a control for the other. Through mass spectrometry, it is possible to assess the presence of D-Amino acids in peptides. Several studies describe that bacteria synthesize common D-Amino acids from peptidoglycan and multiple D-Amino acids in proteins or enzymes, with many other functions [32,33,34]. D-Amino acids are mainly produced by amino acid racemases and also by enzymes that metabolize L-Amino acids, such as cystathionine β-lyase [35].

Therefore, the immunofluorescence microscopy technique can be combined with spectral counting performed at mass spectrometry of viral peptides in bacterial cultures, together with identifying D-amino acids inside the viral peptides of bacterial culture and in the blood of patients.

The aim is to detect SARS-CoV-2 proteins in the gut microbiome cohort of bacterial cultures derived from stool samples [28] and in the plasma of patients positive in nasopharyngeal tests for SARS-CoV-2 versus healthy controls. The genetic aspect of viral replication in bacterial cultures, interactions by electron microscopy and immunofluorescence by light microscopy, evidence of the nitrogen isotope N^15^ introduced into 30-day bacterial cultures, and presence in viral proteins after another seven days have already been described in previous studies [27,28]; which are used as the basis for detailed analysis of genetic–molecular aspects.

Such a combined approach discussed here could better define the bacteriophage behavior, as in this study of the SARS-CoV-2, and, at the same time, expand the knowledge base by an easy-to-use method that can be used in many laboratories—not only for coronaviruses but also for other RNA or DNA viruses (data in preparation).

## 2. Results

In this study, data are presented on the integration of the results of three different tests conducted either in bacterial culture samples in which SARS-CoV-2 was present, derived from fecal samples of infected patients or in bacterial culture samples of healthy individuals, in which SARS-CoV-2-contaminated supernatant, was added. The three tests used were: immunofluorescence microscopy antibodies against SARS-CoV-2 viral proteins, proteomic quantification by mass spectrometry, and identification of D-amino acids within SARS-CoV-2 viral peptides. The connections between prokaryotic cells and viral pathogens are observed in the results, as in the proposed experiments. Fecal samples from healthy and infected individuals were selected for COVID-19. Details of the materials and methods are given in section number 4. Bacterial cultures were performed for up to 30 days, and samples were analyzed by both fluorescence microscopy and mass spectrometry. Electron microscopy represents an internal control.

The analyses were conducted to confirm what has already been observed in previous studies [27,28,36,37,38,39], i.e., the increase in viral proteins within the bacterial cultures, the bacteriophage behavior of SARS-CoV-2 and, in addition, it can be observed that viral proteins with D-amino acids are present in the plasma of COVID-19 patients.

The integration of the data collected through the three tests seems to be applicable in studying the interaction of other human RNA viruses and bacteria derived from the gut microbiome (another study in preparation with another RNA virus).

### 2.1. Immunofluorescence

**Figure 1:**
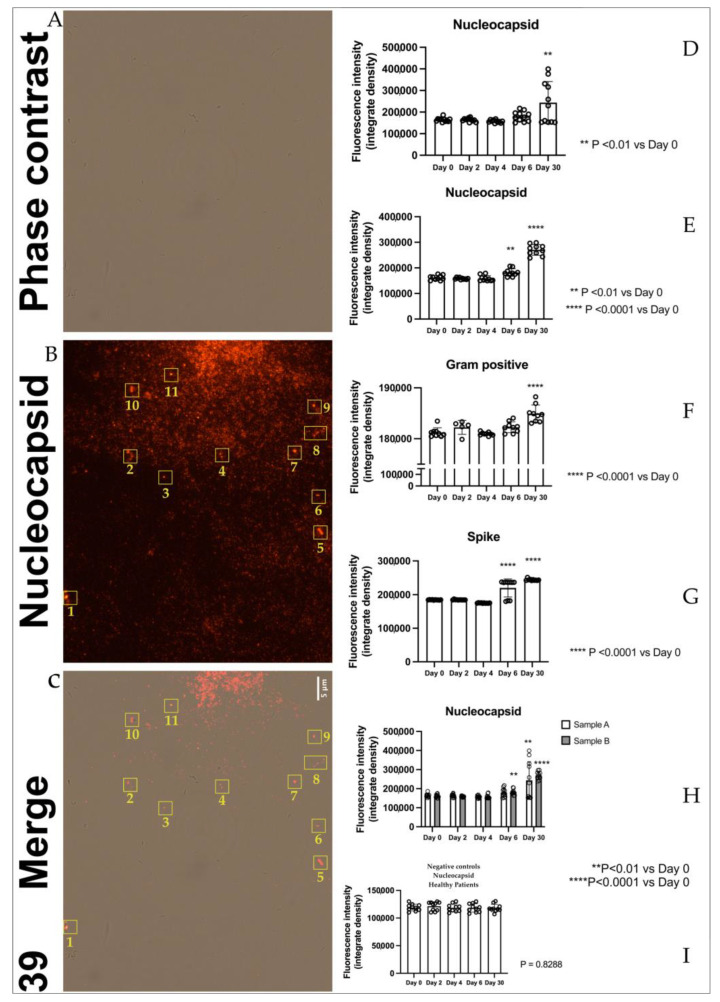
Panel **A:** Bacteriophage behavior of SARS-CoV2 by immunofluorescence. Panel **A–C:** *Dorea formicigenerans* strains (DSM Numbers 3992) infected by SARS-CoV-2. Panel **A** shows the phase contrast image. Panel **B** shows an image of the *Dorea F*. cultures in which supernatant was added with SARS-COV-2, and antibodies against nucleocapsid proteins were detected. Panel **C** shows merged images: the rectangles numbered 1–11 show *Dorea F*. bacteria merging with the signal of nucleocapsid proteins of the virus. Scale bar, 5 μm. Panel **D**: SARS-CoV-2 nucleocapsid protein–fluorescence analysis in samples A_(__1, 2….10)_. The graph shows the quantification of nucleocapsid fluorescence intensity expressed as integrated density within the analyzed samples. ** *p* < 0.01 versus Day 0. Panel **E** SARS-CoV-2 nucleocapsid protein fluorescence analysis in samples B_(1,2,…..10)_. The graph shows the quantification of nucleocapsid fluorescence intensity expressed as integrated density within the analyzed samples. ** *p* < 0.01 **** *p* < 0.0001 versus Day 0. Panel **F**: gram-positive protein fluorescence analysis in samples C _(1,2,…..10)_. The graph shows the quantification of gram-positive fluorescence intensity expressed as integrated density within the analyzed samples. **** *p* < 0.0001 versus Day 0. Panel **G**: SARS-CoV-2 spike protein fluorescence analysis in samples A _(1, 2…..10)_. The graph shows the quantification of spike fluorescence intensity expressed as integrated density within the analyzed samples. **** *p* < 0.0001 versus Day 0. Panel **H**: SARS-CoV-2 nucleocapsid protein fluorescence analysis in samples A _(1, 2…..10)_ and B _(1,2,…..10)_. The graph summarizes the quantification of nucleocapsid fluorescence intensity expressed as integrated density within the analyzed samples. ** *p* < 0.01 **** *p* < 0.0001 versus Day 0. Panel **I**: quantitative analysis of nucleocapsid signal in healthy patients confirms the absence of viral protein or at least a lower signal compared to B _(A+)_ patients.

**Figure 2:**
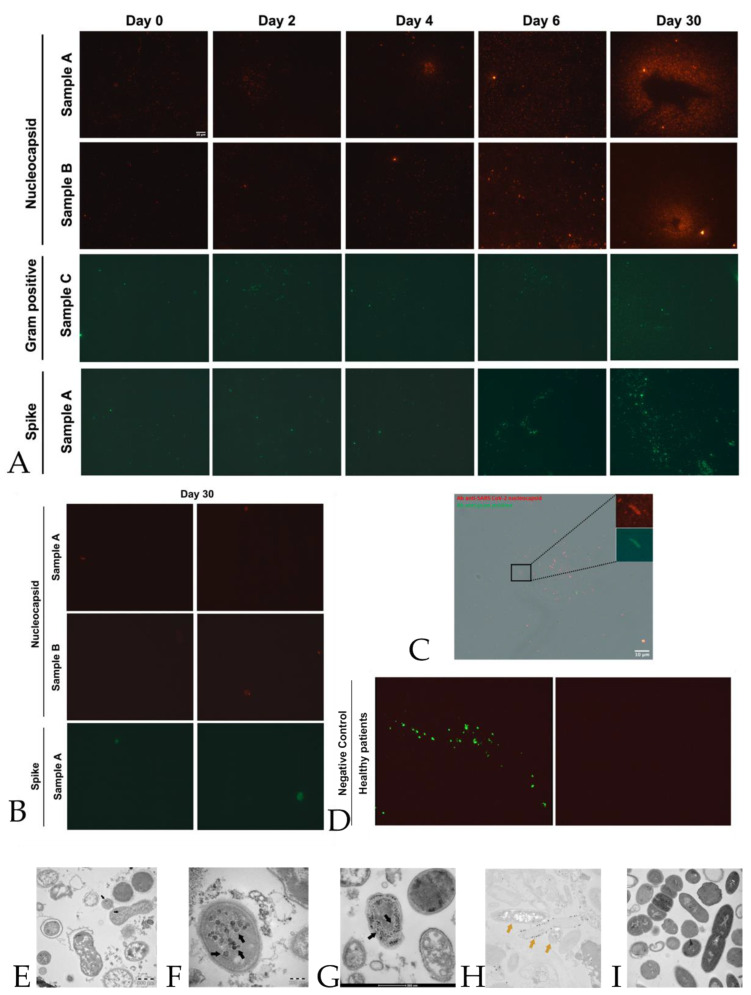
**Quantification of SARS-CoV2 peptides by immunofluorescence**. Panel **A**: Representative panels show immunofluorescence of one sample among the ten samples A, B, and C of nucleocapsid, gram-positive, and spike proteins at 0, 2, 4, 6, and 30 days. Scale bar, 10 μm. All the cases are presented in supplementary materials in Figures S1 and S2. Panel **B:** Negative control of immunofluorescence of samples A and B at day 30 for nucleocapsid and spike proteins. Samples were incubated only with a secondary antibody; subsequently, no protein signal was detected. Panel **C**: A gram-positive bacterial culture from a SARS-CoV-2 positive individual, after 30 days, enlarged and fluorescent to nucleocapsid protein. Scale bar, 10 μ. Panel **D**, left side: shows the negative control immunofluorescence of healthy individuals (negative on nasopharyngeal swab with real-time RT-PCR) with no detection of infected SARS-CoV-2 bacteria (control on proteomic analysis and Luminex assay), as described in Materials and Methods (Section 4). In particular, the merge of gram-positive bacteria (green) and no signal for nucleocapsid proteins (red background) is visible. Panel **D**, the right side, represents the same image as the left side but with only the red background shown. Panel **E**–**G**: Transmission electron microscope images (panels **A** and **B**, TEM FEI, Thermo Fisher Tecnai G2 operating at 120 kV) show SARS-CoV-2 (indicated by black arrows) inside a bacterium. The proteomic profile at mass spectrometry confirms the presence of an abundance of SARS-CoV-2 proteins, together with the Luminex assay test, in the cultures of bacteria under study. Panel **H**,**I**: Immunogold labeling technique at transmission electron microscope on the same samples with anti-nucleocapsid protein of SARS-CoV-2 antibody (golden arrows); the gold particles are inside the bacteria, and no presence in bacteria from healthy individuals (Panel **I**). See more details in Materials and Methods (Section 4).

The immunofluorescence images (panel D Figure 1), obtained with antibodies against nucleocapsid proteins of SARS-CoV-2, present in bacterial cultures derived from stool samples, show the presence of nucleocapsid protein in samples A_(1,2,…..10)_ (derived from stool samples of 10 sick individuals) increased significantly at day 30 compared to day 0. The protein showed a similar trend in samples B _(1,2,…..10)_ (panel E Figure 1) (derived from stool samples of 10 healthy individuals contaminated with 10 supernatants of samples A), where it was raised both at day 6 and day 30, confirming our previous results [28]. Spike protein in samples A_(1,2,…..10)_ (panel G Figure 1) showed a strong increase of signal at day 6 and day 30 in a similar manner as the nucleocapsid protein (panel E Figure 1). The signal of gram-positive protein (panel F Figure 1) on samples C_(1,2,…..10)_ (derived from 10 pellets of samples) increased at day 30, confirming a proliferation of gram-positive bacteria in these samples. Quantitative analysis of nucleocapsid signal in bacteria cultures derived from stools of healthy patients confirms the absence of viral protein or at least a lower signal compared to B _(A+)_ patients (Figure 1 panel I). We have identified, as presented in Figure 1, panels A, B, and C, strains of *Dorea formicigenerans* bacteria (DSM Numbers 3992) labeled with antibodies against nucleocapsid protein of SARS-CoV-2, while in figure panel C, a gram-positive bacterial culture from a SARS-CoV-2 positive individual is highlighted, after 30 days, enlarged and fluorescent to the nucleocapsid protein. In the yellow rectangle, numbered 1–11, Figure 1 panels B and C, bacteria *Dorea formicigenerans* strains are visible (DSM Numbers 3992) and immunofluorescence to antibodies against nucleocapsid proteins of SARS-CoV-2. Figure 2, panel A shows representative immunofluorescence of one sample among the ten samples A, B, and C of nucleocapsid (N), gram-positive, and spike proteins at 0, 2, 4, 6, and 30 days. Negative samples control neg-B (bacteria derived from the gut microbiome of healthy individuals, negative to molecular oropharyngeal swab test, (RT-PCR analysis, Viasure real-time PCR detection kit, Cortest Biotec), and molecular feces test, are shown in panel D of Figure 2, where antibodies versus gram-positive bacteria and nucleocapsid proteins were introduced in the samples—with no signals present in the samples of healthy controls (Figure 2, panel D, left side). The other negative control was also performed with only secondary antibodies, with the absence of immunofluorescence visible in panel D, Figure 2, seen on the right. All the cases are presented in supplementary materials in Figures S1 and S2.

### 2.2. Spectral Counting Quantification Peptides of SARS-CoV-2 at Mass Spectrometry Analysis

**Figure 3:**
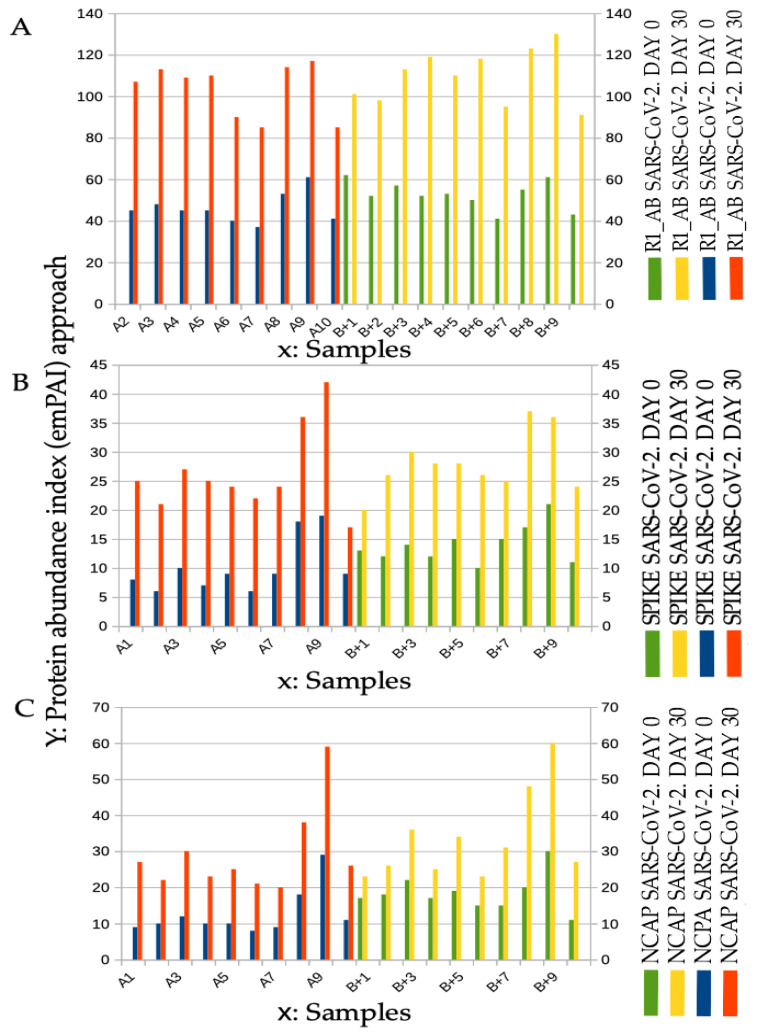
Increase of SARS-CoV-2 protein abundance at spectral counting quantification at day 0 and day 30 in the samples called A and in the samples called B+ (+ Contaminated with A). Panel **A**: Protein R1_AB SARS-CoV-2 spectral counting quantification. The spectral counting confirms the growth of the abundance of the peptides. *“Replicase polyprotein 1AB (P0DTD1, UniProt) is a multifunctional protein involved in the transcription and replication of viral RNAs”.* Panel **B**: Protein SPIKE SARS-CoV-2 spectral counting quantification). The spectral counting confirms the growth of the abundance of the peptides. *“Spike protein S1 (P0DTC2 UniProt) attaches the virion to the cell membrane of the epithelium cells by interacting with host receptor.”* [database UniProt]. Panel **C**: Protein NCAP SARS-CoV-2 spectral counting quantification. The spectral counting confirms the growth of the abundance of the peptides. “*Nucleocapsid protein (P0DTC9-UniProt) packages the positive strand viral genome RNA into a helical ribonucleocapsid (RNP) and plays a fundamental role during virion assembly through its interactions with the viral genome and membrane protein M. plays an important role in enhancing the efficiency of subgenomic viral RNA transcription as well as viral replication”.*

Spectral counting (Figure 3) is a semiquantitative mass spectrometry approach for defining the abundance of the molecules under study, and it was obtained using the exponentially modified protein abundance index (emPAI) approach: “*Absolute amount determination without added standards is based on the assumption that proteins detected make up most of the protein content in the sample and unidentified proteins are at low abundance and have only minor effect on the total protein amount. This simplification has been used successfully for absolute protein expression (APEX), Total Protein Approach (TPA), and emPAI. Such simplification has resulted in good agreement with a spike in standard protein concentrations.”* A recently published study has compared different label-free absolute quantification strategies and is an excellent reference point, where large-scale proteome quantification is made with or without standard proteins, referred to as the calibration curve model and the direct proportionality model, respectively [30]. Spectral counting was performed in every aliquot, considering the abundance of the SARS-CoV-2 peptide (Figure 3). The infected samples were compared with the samples control. The normalized value was obtained by using the formula (i): Nf = C * Stc(i)/Stc(c). Nf is the normalized value, weight *C* is a coefficient calculated on the basis of the false discovery rate, *Stc(i)* is the protein statistical counting coefficient obtained by analyzing the infected samples, and *Stc(c)* is the statistical counting coefficient obtained from the samples control.

### 2.3. D-Amino Acid Presence at Mass Spectrometry in Plasma

**Figure 4:**
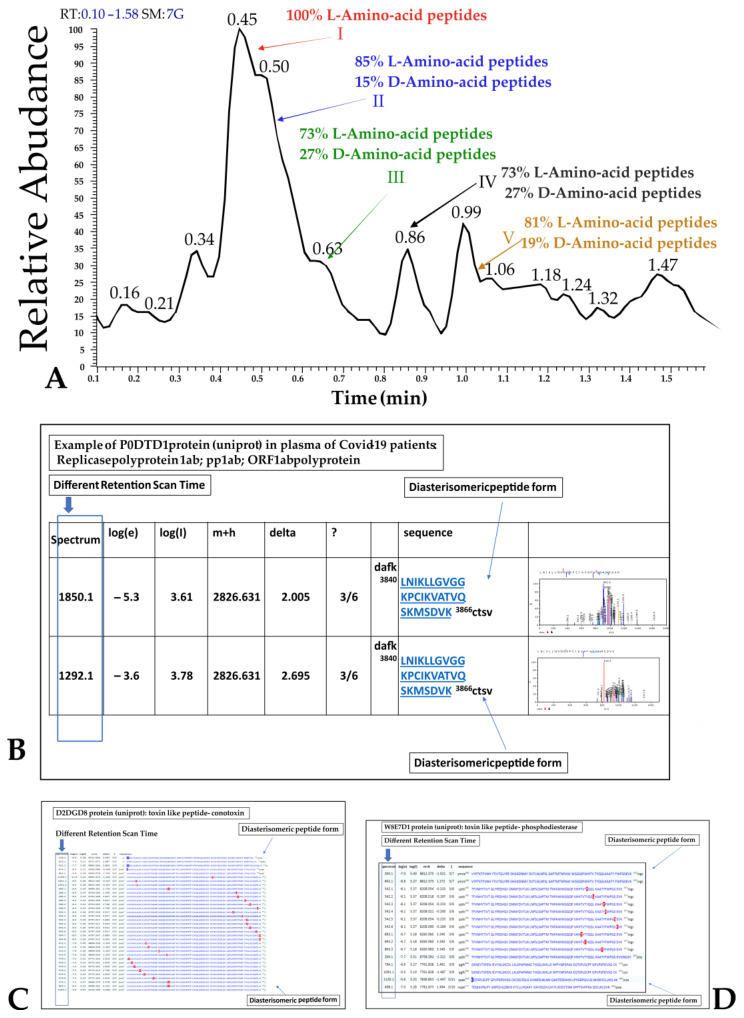
Representative image of diastereomeric peptides containing D-Amino acid in SARS-CoV-2 proteins and in toxin-like peptides in blood samples of infected individuals. Panel **A**: SACI-CIMS scan of voltages. Selective retention time ion cutting was employed; Panel **B**: Example of P0DTD1 protein (UniProt) in plasma of COVID-19 individuals. Replicase polyprotein 1ab; pp1ab; ORF1ab polyprotein. The rectangles show different retention scan times, suggestive of different D-Amino acids presence. Images of the spectra on the right side of panel B are highlighted at higher resolution in Figure S3 of supplementary materials. Panel **C**: different retention scan times of toxin-like peptides, D2DGD8 protein (UniProt): toxin-like peptides conotoxin. Panel **D**: different retention scan times of toxin-like peptide W8E7D1 protein (UniProt): toxin-like peptides phosphodiesterase.

Diastereomeric peptides containing D-Amino acids (Figure 4) have been identified in all COVID-19 individuals and not in the healthy control using the multifragmentation pathway approach at different mass spectra retention times [40,41]. The enantiomeric peptide form has been resolved by means of liquid chromatography using a HOLO 50x2.1 2.7 µm (VWR, Milan, Italy) particle size column. The repeated peptide sequences exhibiting the fragmentation pathways identified by the database search approach at multiple retention times have been marked as enantiomeric formations containing D-Amino acid. D-structure was identified by specific fragmentation pathways [42]. Representative images of these observations are presented in Figure 4. Particular interest is the different retention scan times of the same peptides (rectangular violet in Figure 4 panel B–D). 

The finding of SARS-CoV-2 proteins discussed in this study and toxin-like peptides, as we have already described [37,38], characterized by the presence of D-Amino acids, is scientifically intriguing and highlights that viral proteins in the plasma of sick patients could be derived from bacterial production.

### 2.4. Electron Microscopy

As an internal control, we performed electron microscopy in accordance with the current literature asserting that in the case of COVID-19 diagnosis, the use of image analysis to confirm the presence of SARS-CoV-2 particles complements the detection of molecular traces of SARS-CoV-2 specific proteins or nucleic acids (and vice versa). Analysis performed by transmission electron microscopy showed virus-like particles within the bacteria, as can be seen in Figure 2, panels E–I, using immunolabelling techniques. No mammalian eukaryotic cells are present after 30 days of bacterial cultures.

## 3. Discussion

Some studies have reported clues as to the involvement of bacteria in the pathogenesis of SARS-CoV-2. An American group showed that there is a correlation between gastrointestinal symptoms and the persistence of viral RNA for up to 7 months in 3.8% of the subjects tested after the initial manifestation of the disease [43]. In the autumn of 2021, biological samples were collected from Norwegian rat (Rattus Norvegicus) carcasses in New York City. Samples were collected from the wastewater system. SARS-CoV-2 viral RNA was found in them [44]. It is now well established in the literature that both species jump and species regression occurs in SARS-CoV-2 transmission. These data cannot conceal doubt about bacterial involvement in spreading and transmitting SARS-CoV-2. In fact, despite countless efforts of the vaccination campaign, there are cases of severe COVID-19 disease in the unvaccinated group and some in the vaccinated group [45,46].

In a case report presented in [45], the presence of the virus at autopsy in almost all organs of the deceased except the liver and olfactory bulb was observed. The elderly man was hospitalized with profuse diarrhea but had negative oropharyngeal swabs for SARS-CoV-2. The unvaccinated roommate tested positive for the virus six days later. This mode of infection, most likely oral–fecal, complements [28,47], who noticed how the oral–fecal route of infection is just as important as the respiratory route, as we have noted in [28]. The same authors [47] mention how the gastrointestinal tract is rich in cells with receptors for angiotensin-converting enzyme 2 (ACE2) and emphasize how important it is to consider that the virus also replicates in this anatomical district. In our previous study [48], it was observed that a patient admitted with COVID-19 pneumonia was negative in six multiple nasopharyngeal tests while testing positive in the molecular real-time qRT-PCR test on a stool sample. After 18 months, he became infected again, despite his high antibody titer, but was now positive on nasopharyngeal testing [49].

Authors in [50] suggested a new approach for detecting viruses in fecal samples and showed how they could be found in such samples. The study presented in [2] also described how fecal samples were collected and explained how the finding in feces also occurred for SARS and MERS. In a recent retrospective cohort study of 46 children tested, 57% still had fecal test positivity after ten days of symptomatic recovery, despite negativity on oral–nasopharyngeal tests [51]. Therefore, authors in [3] recommend oral–fecal viral testing during hospitalization and in the final stage of disease recovery.

Petrillo et al. [27], in bacterial cultures from fecal samples of COVID-19 patients, investigated how it is possible to assess the viral load and the interactions between the virus and bacterial community. With the same test, the authors also verified oral–fecal transmission, as SARS-CoV-2 positive samples contaminated samples with bacteria that were initially negative for the same virus.

Some studies highlighted the essential interaction of the immune system with the human microbiome and noted how its bacteriological characterization changes according to sex, race, age, geolocation, and the presence of disease severity, such as COVID-19 [52,53]. The bacterial co-factor appears to play a critical role in SARS-CoV-2 infection, and many point out that the gut microbiome is disrupted [28,54]. Previously, some studies highlighted the bacterial co-factor with another RNA viral infection [55,56]. The correlation with gram-positive bacterial flora highlights the complexity of viral infections [57,58,59]. The human epithelial mucosa, human subepithelial lymphatic tissue, and the bacteria of the human microbiome appear interconnected, and it becomes crucial to study aspects that are still uncertain. For example, one might also consider that the plasma concentration of CD4, CD8, and CD3 lymphocytes that tend to decrease in this viral infection, but also in others, is potentially evident because of the increased migration of these cells at the gut epithelium–microbiota interface. These connections begin to be evident during viruses’ general defense, invasion, and replication phases [60,61]. In fact, reviewing the existing literature on HIV [62,63,64,65,66,67] and poliovirus [68], other RNA viruses also appear to have significant involvement in the gut microbiome. In fact, a recent study observed HIV gene sequences in bacteria isolated from the oropharyngeal tract of Kenyan children [63], and this finding is synergic with the diatribe of the bacterial cofactor in HIV and its connection with mycoplasmas [66]. On the other hand, poliovirus (PLV) (another RNA virus of a little more than 7000 bases) has long been considered respiratory until the publications by Dr. Sabin [68,69,70,71] appeared, in which the intestinal replication of poliovirus, the absence of replication in oral vaccine vaccinees and the persistence of replication in injection vaccinees were demonstrated. These publications changed the endpoint of vaccination and obtained major effects. Many years later, a group of scholars analyzed that the PLV used for the oral vaccine had as many as 57 mutations compared to the aggressive strain of poliovirus [72]. History reminds us that poliovirus has affected Western communities for centuries, and we do not know how many mutations have occurred. On the other hand, Petrillo et al. [27], at the time of analysis of bacterial samples, highlighted the mutations of SARS-CoV-2 in bacterial cultures and introduced mutations that were not yet present in the various Western countries. There seems to be increasing emergence throughout the history of wall-to-wall help from bacteria in the genesis of symbiotic mutations for the host.

In this study, the presence of the nucleocapsid and spike proteins of SARS-CoV-2 was observed from day 0 to day 30 using fluorescence microscopy analysis. In addition, an increase in the fluorescence of the spike proteins was observed, confirming the bacteriophage behavior of SARS-CoV-2, similar to what has been discussed previously [27,28]. In addition, it is confirmed that the virus appears to interact with prokaryotic cells [28] of the human mucosal microbiota, stimulating them to produce toxins [37,38] and modifying the property of the virus to interact with epithelial cells, especially with bacteria in the microbiome. The authors in [28] showed that during SARS-CoV-2 infection, the presence of gram-positive bacteria increases significantly. This fact correlates with the increased mortality of patients. At the same time, the work presented in [27] also showed in vitro tests in bacterial cultures in which SARS-CoV-2 is present and that some antibiotics act against the microbiota—leading to a decrease in viral RNA load in samples. This demonstrates the need to induce resistance in bacteria affected by the viral pathogen.

What was not suspected is that SARS-CoV-2 proteins detected in the plasma of infected patients, as shown in the tests, may contain many D-Amino acids. The presence of D-Amino acids within any protein is historically dated and considered a bacterial activity in the constitution of the protein itself [32,33,34,35]. This finding opens another scenario of immunopathology for its versatility in being easily reproduced.

Immunofluorescence microscopy allows visualization of the binding of a recognized antibody to a specific protein. It also can be used empirically to quantify the increase in viral protein load over time. The data can be integrated with microscopic immunofluorescence using mass spectrometry and the following semi-quantitative spectral counting analysis [30]. In fact, spectral counting allows for a semi-quantitative assessment of protein abundance in the sample and evaluation over time in bacterial cultures, as demonstrated in this study. At this point, a final therapeutic consideration on a logical basis needs to be proposed. It is historically known that bacteria are adept at protecting themselves with resistance to antibiotics, and one of the ways of doing this is to continue to combine antibiotics with cultures. It should not be excluded that bacteria may also be adept at inducing resistance against viral pathogens and, in this case, against SARS-CoV-2. Therefore, analyzing those who have fallen ill more than once would be necessary. The interaction of genetic makeup is simpler than the interaction with a synthetic molecule such as an antibiotic. The question which remains is: how long does it take for bacteria to generate resistance to SARS-CoV-2? Once this is solved, we believe it will be possible to plan vaccines based on probiotics that are resistant to the virus.

## 4. Materials and Methods

### 4.1. Virus Growth in Bacterial Cultures and Sample Preparation

#### 4.1.1. Collection and Storage of Fecal Samples

Fecal samples (feces) were obtained from 10 confirmed COVID-19 patients, confirmed by an oropharyngeal molecular test positivity (Easy^®^ SARS-CoV-2) for SARS-CoV-2 (samples called A_1,2,3,4,5,6,7,8,9,10_) and ten fecal samples obtained from healthy individuals, negative to oropharyngeal molecular testing for SARS-CoV-2 and never sick for COVID-19 (samples called neg-B). The fecal samples were stored at 4 °C until processing. Informed consent from patients was obtained according to local legislation.

#### 4.1.2. Initial Stool Samples A and neg-B Processing

Homogenized fecal material was obtained by mixing it for 30 min in a pneumatic mixer (MiraclePaintSportDC-1-C, Minneapolis, MN, USA) with sterile distilled water (4 °C) in a proportion of 1.5 times the weight of a fraction of picked up sample.

Homogenized fecal materials were washed in 10 mL of NaHCO3 50 mmol (from Sigma Aldrich), pH 7.8, and centrifuged at 1500× *g* for 10 min. Pellets were then re-suspended in 10 mL NaCl 10 mmol. The aliquots of suspensions were placed in tubes (5 mL) with growing broth (NutriSelect™ Plus nutrient broth No. 70122-500G, Sigma-Aldrich) in an orbital shaker at 10 g at the optimal growing temperature of 37 °C for one week.

#### 4.1.3. Medium Preparation

The cultures of samples A and samples neg-B were grown in NutriSelect™ Plus nutrient broth (No. 70122-500G, Sigma-Aldrich), suitable for multi-bacterial growth. Following the protocol recommended by the supplier, the medium was prepared as follows: 25 g was dissolved in 1 L of double-distilled water and filled into test tubes. The test tubes were autoclaved at 121 °C for 15 min. All steps were conducted at a temperature below 8 °C, protected from direct light. The final composition of the culture medium was Peptone (15 g/L), yeast extract (3.0 g/L), sodium chloride (6.0 g/L), D (+)-Glucose (1.0 g/L), pH 7.5 at 25 °C.

#### 4.1.4. Bacterial Growth

The tubes with the culture broth and bacteria were placed in a 10 G orbital shaker at the optimal growth temperature of 37 °C, and the liquid culture was allowed to grow progressively. Bacterial growth was monitored by measuring the optical density (OD) with a microplate absorbance reader (spectrophotometer).

#### 4.1.5. Nucleic Acid Extraction

Total nucleic acid extraction was performed using the NucliSens^®^ easyMAGTM extraction system (bioMerieux, Marcyl’Étoile, Lyon, France) according to the manufacturer’s instructions.

#### 4.1.6. Evaluation of SARS-CoV-2 RNA Load

Luminex technology (Life Technology, Austin, Texas, USA) was used to detect the viral RNA load in bacterial cultures. Aliquots of samples to be measured were always centrifuged at 1500× *g* for 10 min, and the supernatant was taken for the measurements. The detection was performed by using NxTAG^®^ CoV Extended Panel, a real-time reverse transcriptase PCR assay detecting three SARS-CoV-2 genes on the MAGPIX^®^ NxTAG-enabled System MAGPIX instrument and the AccuPlexTM SARS-CoV-2 Reference Material Kit (SeraCare) as reference standard with sequences from the SARS-CoV-2 genome.

Multiplex plates were produced in-house, and RNA tags were added before the analysis in agreement with manufacturer instructions. Multiplex plates were transferred to a MAGPIX heater pre-heated to 37 °C. The signal acquisition was performed using the xPONENT and SYNCT software (Luminex Molecular Diagnostics) using a commercially available reference standard with sequences from the SARS-CoV-2 genome (AccuPlexTM SARS-CoV-2 Reference Material Kit, SeraCare). Each running batch handled up to 94 clinical samples, plus the positive and negative controls performed with the kit under manufacturer instructions. The total turnaround time was around 4 h. Luminex detection was reported in arbitrary units (AU- determined by using the PROSAD methodology described in [73].

#### 4.1.7. Creation of Samples B and C

After one week of culture, ten supernatant aliquots (0.5 mL) of each sample A, containing a viral RNA load tested as reported above, centrifuged at 13,000× *g* for 15 min, were inoculated into 5 mL of each sample B to generate Sample B(_A+_). Samples C were obtained by resuspending the residue pellet obtained by centrifugation of the aliquot of sample A.

All samples: sample B(_A1+, A2+, …A10+_), samples C(_1,2,…..10_), and equivalent aliquots of samples A(_1,2,…..10_) and neg-B(_1,2,…..10_), were grown in an orbital shaker at 10 g at 37 °C for 30 days. Aliquots were taken at days 0, 2, 4, 6, and 30 following the date of inoculation (day 0). Samples neg-B are the cultures of stool bacteria of healthy individuals that are the negative control.

#### 4.1.8. Supernatant Collection

On initial day 0 and days 2, 4, 6, and 30, 0.20 mL of supernatant was collected from each culture of the samples called A_(1,2,…..10)_, B_(1, 2, …10)_, C _(1,2,…..10)_, neg-B_(1,2,…..10)_ (negative control of bacteria derived from the fecal matter of healthy people) for the immunofluorescence, and at days 0 and 30, 0,20 mL of supernatant was collected from each culture of the sample called A_(1,2,…..10)_, B_(1, 2, …10),_ neg-B _(1,2,…..10)_ for the proteomic profile and spectral counting [30]. Each collection was followed by adding an equal amount (0.20 mL) of previously prepared sterile broth.

#### 4.1.9. *Dorea formicigenerans* Strains Growth

*Dorea formicigenerans* strains were purchased at the Leibniz Institute DSMZ-German Collection of Microorganisms and Cell Cultures (DSM Numbers 3992). It was grown in liquid broth according to the information provided by the Leibniz Institute DSMZ: chopped meat medium, with 1% glucose, anaerobic, 37 °C. After 7 days, was inoculated with the ultrafiltrate of the supernatant of the initially SARS-CoV-2 positive bacterial culture using the appropriate Millipore microfilter, UFC701008, Centricon Plus-70 Centrifugal Filter. 48 h later, 0.20 mL was collected from the *Dorea F*. (3992) liquid culture and placed on loaded slides.

#### 4.1.10. Plasma Collection [37]

In this study, it was possible to extend the data by searching and observing the proteomic profile of D-Amino acid presence in viral (SARS-CoV-2) peptides of our previous papers [37,38]. Plasma samples were collected from 20 COVID-19-positive patients and from 10 healthy individuals (control). Each individual expressed free and informed written consent according to current legislation.

### 4.2. Immunofluorescence

The aliquot of supernatant of each culture was placed on positively charged slides (Superfrost Plus, Ref J1800AMNZ, Thermo Scientific). The slices were fixed with 4% *paraformaldehyde* (PFA) for 5 min. After permeabilization with Triton 0.3% in phosphate-buffered saline (PBS) for 10 min, sections were rinsed in PBS and blocked with bovine serum albumin (BSA) 1% and saponin 0.05% for 30 min. The slices were then incubated with primary antibodies for 2 h at room temperature. Secondary antibodies were incubated for 1 h at room temperature. The following primary antibodies were used: rabbit polyclonal anti-SARS-CoV-2 nucleocapsid protein antibody (1:500 dilution, Rockland, n. 200-401-A50), mouse monoclonal anti-SARS-CoV/SARS CoV-2 spike antibody (1:500 dilution, GeneTex, n. GTX632604), mouse monoclonal gram-positive bacteria antibody (1:500 dilution, GeneTex, n. GTX42630). The following secondary antibodies were used: goat anti-rabbit CY3 (1:600 dilution, Invitrogen, #A10520), goat anti-mouse Alexa Fluor 488 (1:600 dilution, Invitrogen, #A28175). The stained slices were then assembled with mounting medium (Dako, CA, United States). Some negative controls were incubated with three times higher concentrations of nucleocapsid antibody than others to be sure to detect, or not, the protein signal.

The Zeiss Axioplan 2 color microscope, Axiocam 305 (Zeiss, Jena, Germany), was used to acquire fluorescent images. Immunofluorescence experiments were carried out on a total of 40 slices, derived from 30 SARS-COV-2 positive bacterial cultures (samples A, B, and C) and 10 bacterial cultures of healthy subjects (samples neg-B). Moreover, immunofluorescence images were obtained from *Dorea F*. culture contaminated with the supernatant filtrate of SARS-CoV-2 positive cultures.

The negative control of the bacterial cultures was performed without primary antibodies versus gram-positive bacteria and without primary antibodies versus nucleocapsid and spike SARS-CoV-2 proteins (Figure 2B). The second negative control was obtained by adding antibodies versus gram-positive bacteria and nucleocapsid proteins in the bacteria culture derived from healthy persons negative to the oropharyngeal swab test and Luminex assay test previously performed in the same culture (Figure 2 Panel D and supplementary materials Figure S1). It was assumed that the control of immunogenicity of antibodies versus gram-positive bacteria is reflected in studies [74,75]. The control of immunogenicity of primary antibodies versus the nucleocapsid protein of SARS-CoV-2 was assumed from [76].

### 4.3. Proteomic Profiling Analysis of Bacteria Cultures for Spectral Counting and for D-Amino Acid Presence Observation

#### 4.3.1. Preparation of Buffers for Each Supernatant of Bacterial Culture Aliquot

*Ammonium Bicarbonate (Digestion Buffer) 50 mmol pH 7.8.* Preparation procedure: weigh into a weighing pan 0.4 g of ammonium bicarbonate (NH4HCO3)(Sigma Aldrich). Pour the powder into a 100 mL bottle and add 100 mL (measured with the measuring cylinder) of double-distilled water. The bottle was shacked until the NH4HCO3 was completely dissolved.

#### 4.3.2. Preparation of Reagents

*Trypsin solution 25 ng/μL.* Preparation procedure: resuspend in a vial 20 μg of solid trypsin (Sigma Aldrich) in 800 μL of the 50 mM NH4HCO3 solution. Vortex the vial until trypsin is completely dissolved.

#### 4.3.3. Liquid Chromatography Surface-Activated Chemical Ionization—Cloud Ion Mobility Mass Spectrometry (LC-SACI-CIMS) Instrumentation [77,78,79,80]

The analysis was performed using an HPLC Surveyor (Thermofisher, USA). The column used was a Halo Peptide ES-C18, 2.1 × 50 mm, 2.7 µm. Analyses were performed using a two-phase gradient: Phase A (H2O + 0.2 % Formic acid (HCOOH)) and Phase C acetonitrile (CH3CN). The chromatographic gradient used is shown in Table 1. The volume of the sample injected is 5 µL. Data acquisition was performed using Surface Activated Chemical Ionization—Electrospray—NIST Bayesian model search (SANIST-CIMS), a ‘SANIST’ mass spectrometer.

The results of this analysis were processed using ‘SANIST-Hb’ software [78], analyzing three different databases UniProt 3 containing, respectively, proteins (Table 2) to SARS-CoV-2 and generic toxins (Toxins), respectively, (data in preparation for toxins results).

#### 4.3.4. Plasma Preparation

Each plasma sample was treated as follows: 5 µL of trypsin was added to 50 µL of plasma and vortexed for one minute. The procedure was repeated 10 times. Then the sample was diluted in 950 µL of NH_4_HCO_3_ 50 mmol and enzymatically for 72 h. The solution was centrifuged at 13,000× *g* for 10 min. A total of 100 µL of the supernatant was collected and spiked with 1 µL of formic acid analyzed. The obtained solution was analyzed through liquid chromatography—surface-activated chemical-ionization-Cloud ion mobility mass spectrometry (LC-SACI-CIMS) [77,78,79,80].

### 4.4. Internal Control with Electron Microscopy, Figure 2-Panel E–G

Electron microscopy for the samples was performed as an internal control, and the images are presented in Figure 2, panel E–G.

Aliquots of bacterial pellets (0.5 mL) obtained after 30 days of bacterial cultures, positive to molecular test for SARS-CoV-2, were fixed by resuspending them in 1% glutaraldehyde. They were then centrifuged at 10,000 rpm for ten minutes, harvested as pellets, fixed in 2% OsO4 and processed according to the standard procedures, dehydrated in ethanol followed by infiltration and embedded in Epon resin and polymerized at 60 °C for 48 h. Then, 60-nm thin sections were cut from the sample using a Reichert-Jung ultramicrotome. Grids were counterstained with UranyLess EM stain and lead citrate 3% (Electron Microscopy Science). Images were obtained from thin sections under an electron microscope (Tecnai G2 Spirit BioTwin; FEI, Hillsboro, OR 97124-5793 USA) equipped with a VELETTA CCD digital camera (Soft Imaging Systems GmbH, Johann-Krane-Weg 39, 48149 Münster, Germany). The protocol was performed three times in three different facilities with different operators.

#### Immunogold Labelling Technique, Figure 2-Panel E–G

Bacterial pellets (0.5 mL) were fixed by resuspending with a mixture of 0.05 glutaraldehyde of 4% paraformaldehyde in 0.2 M HEPES buffer. Then bacteria pellets, obtained by centrifugation (10,000 rpm for ten minutes), were resuspended, washed with PBS, and incubated first in blocking solution (50 mM NH4Cl, 0.1% saponin, 1%BSA in PBS) and then in primary antibody against Nucleocapsid SARS-CoV-2 (N) protein (Abcam, #ab273167). Then the cells were washed with PBS and incubated with NANOGOLD conjugated anti-rabbit Fab fragments (Nanoprobes, #2004). After washing in PBS and distilled water, bacterial pellets were kept in the gold-enhancement mixture (Nanoprobes). Immunolabelled specimens were post-fixed in OsO4 and uranyl acetate and embedded in EPON. Thin sections were cut from embedded specimens using Leica EM UC7 (Leica Microsystems). Electron microscopy images were acquired from thin sections under an electron microscope (Tecnai G2 Spirit BioTwin; FEI) equipped with a VELETTA CCD digital camera (Soft Imaging Systems GmbH).

The first negative control was obtained by omitting the primary antibody and using only the secondary gold-conjugate antibody. The control of immunogenicity of antibodies primary versus nucleocapsid protein of SARS-CoV-2 was assumed from [76].

### 4.5. Statistical Immunofluorescence Analysis

Graphs and data analysis were performed using Graph Pad Prism 9 software. The average fluorescence from at least image (Figure 1 and Figure 2) per each sample was measured as integrated density on ImageJ software for analysis. Values are expressed as mean ± SEM. Statistical analysis was performed by one-way ANOVA comparing the average values of each sample. *p* values < 0.05 were considered statistically significant.

## 5. Conclusions

In this study, a combined approach, consisting of immunofluorescence and spectral counting at mass spectrometry, is presented to highlight the initial behavior of the new pathogen, such as SARS-CoV-2, on the human microbiome. It was demonstrated that the immunofluorescence microscopy technique could be combined with spectral counting performed at mass spectrometry of viral peptides in bacterial cultures to identify D-Amino acid presence inside the viral peptides of bacterial culture and in the blood of patients.

Immunofluorescence and spectral counting at mass spectrometry applied to the method proposed in [27] seems to facilitate the detection of viral proteins in the samples examined and allow the observation in an in vitro study of any abundance of viral proteins produced during bacterial culture. It is important to highlight the bacteriophage behavior of SARS-CoV-2 but also of other viruses (data in preparation). Increasing signal intensity by fluorescence microscopy using fluorescent antibodies in fractions of bacterial cultures with the virus under study—conducted up to 30 days—appears to be increasingly important for ignoring possible bacteriophage behavior. New considerations on the presence of D-Amino acid inside proteins of SARS-CoV-2 and toxin-like peptides detected in the plasma of COVID-19 patients could be valuable for understanding the virology mechanism of RNA viruses that can bind and interact both with epithelial cells and bacteria of the human gut microbiome.

The experiments and findings presented to enhance a general understanding of the interactions between the human microbiota and virus RNA connections. It is recommended that bacterial cell cultures of fecal samples from virus-infected individuals be used and that the increase in viral proteins be observed with a fluorescence microscope and spectral counting at mass spectrometry for up to 30 days of culture or longer in order to observe for diagnostic purposes, whether the pathogen has bacteriophagic behavior. Understanding whether some viruses have bacteriophagic behavior allows vaccine therapies to be focused either on certain toxins produced by bacteria in the microbiome or on finding inert or symbiotic viral mutations in the human gut microbiome.

Moreover, it is advisable for health authorities to consider health policies that would include personal hygiene measures aimed at immediately reducing the microbial load in food and used water. Eradication of secondary bacterial infections would add extra benefits and contribute to preventing a current epidemic.

The bacterial mechanism for the virus shown or for possible other viruses opens new preventive, vaccine, and therapeutic scenarios.

## Figures and Tables

**Table 1:** Chromatographic gradient.

Time (Minutes)	%C	Flow mL/min
0	2	0.250
2.5	2	0.250
3	80	0.250
7	80	0.250
8	2	0.250
15	2	0.250

**Table 2:** Uni-Prot 3 proteins of SARS-CoV-2, checked in proteomic analysis, using ‘SANIST-Hb’ software [78].

ID Protein	Full Name
P0DTD1	R1AB_SARS2 Replicase polyprotein 1ab[…] rep, 1a-1b Severe acute respiratory syndrome coronavirus 2 (2019-nCoV) (SARS-CoV-2)
P0DTC9	NCAP_SARS2 Nucleoprotein[...] N Severe acute respiratory syndrome coronavirus 2 (2019-nCoV) (SARS-CoV-2)
P0DTC2	SPIKE_SARS2 Spike glycoprotein[...] S, 2 Severe acute respiratory syndrome Coronavirus 2 (2019-nCoV) (SARS-CoV-2)

## Data Availability

Not applicable.

## Supplementary Materials

The following supporting information can be downloaded at: https://www.mdpi.com/article/10.3390/ijms24043929/s1.
