# Supplementary material for: Analysis of Bacteriophage Behavior of a Human RNA Virus, SARS-CoV-2, through the Integrated Approach of Immunofluorescence Microscopy, Proteomics and D-Amino Acid Quantification
Source: Int J Mol Sci. 2023 Feb 15;24(4):3929. doi: 10.3390/ijms24043929 (PMC9965620; doi:10.3390/ijms24043929)
Supplement: Supplementary file 1 [file ijms-24-03929-s001.zip › ijms-2135414-supplementary materials.pdf]

Figures S1 and S2 of the supplementary materials (sm) show the tests performed, including the controls (Figure S1, panel A)

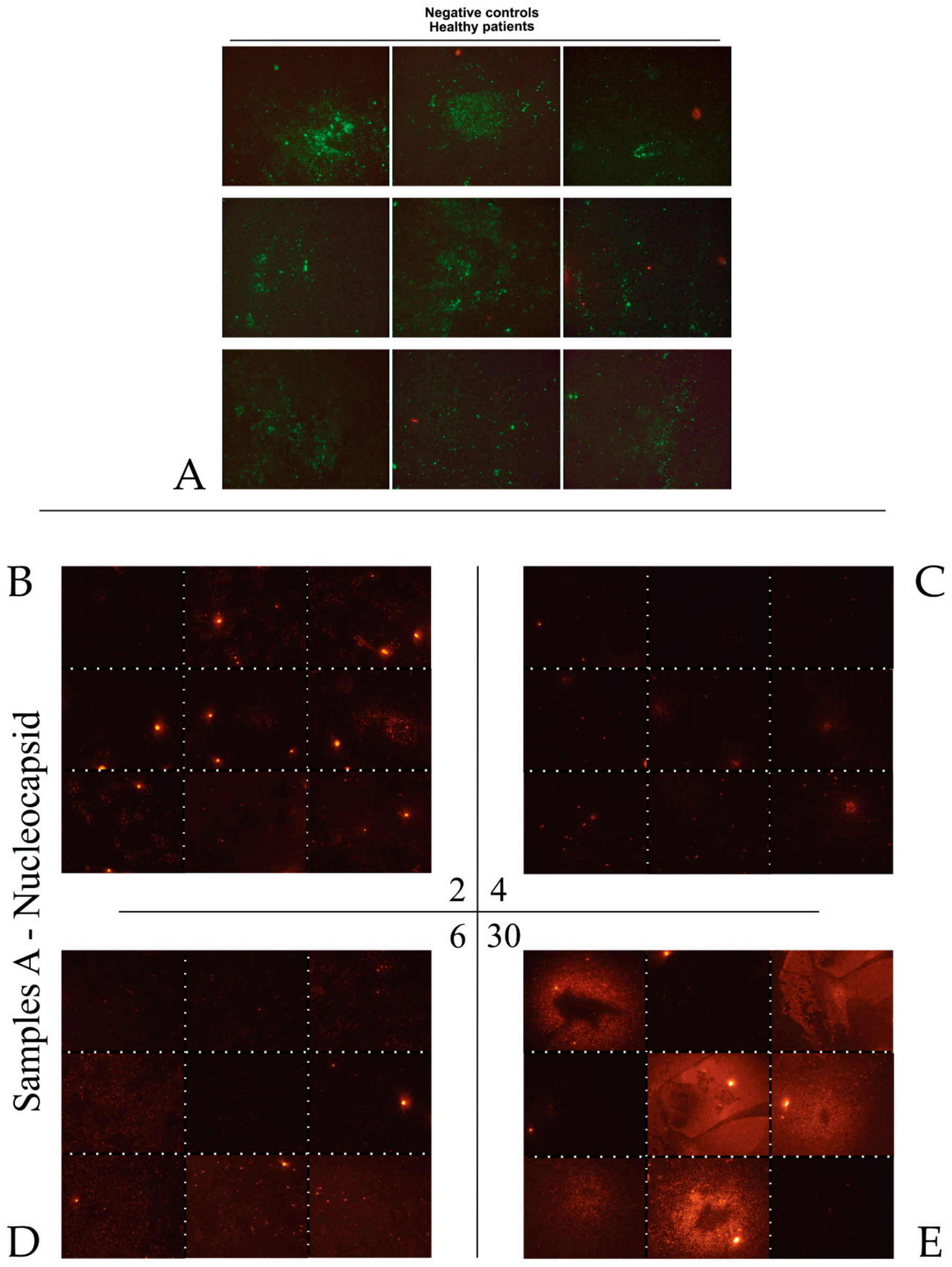

Figure S1. (supplementary materials, upper part) shows the negative controls of 9 samples (neg-B) from healthy patients (Panel A) of the merge signal of gram-positive bacteria (green) and no signal for nucleocapsid proteins (red background). The bottom part shows samples A (Panels B, C, D, E) with increased nucleocapsid protein signals up to the 30th day of bacterial culture. **Panel A**, samples derived from 30 days culture of healthy patients. **Panel B**, samples A from cultures after two days. **Panel C**, samples A from cultures after four days. **Panel D**, samples A from cultures after 6 days. **Panel E**, samples A from cultures after 30 days.

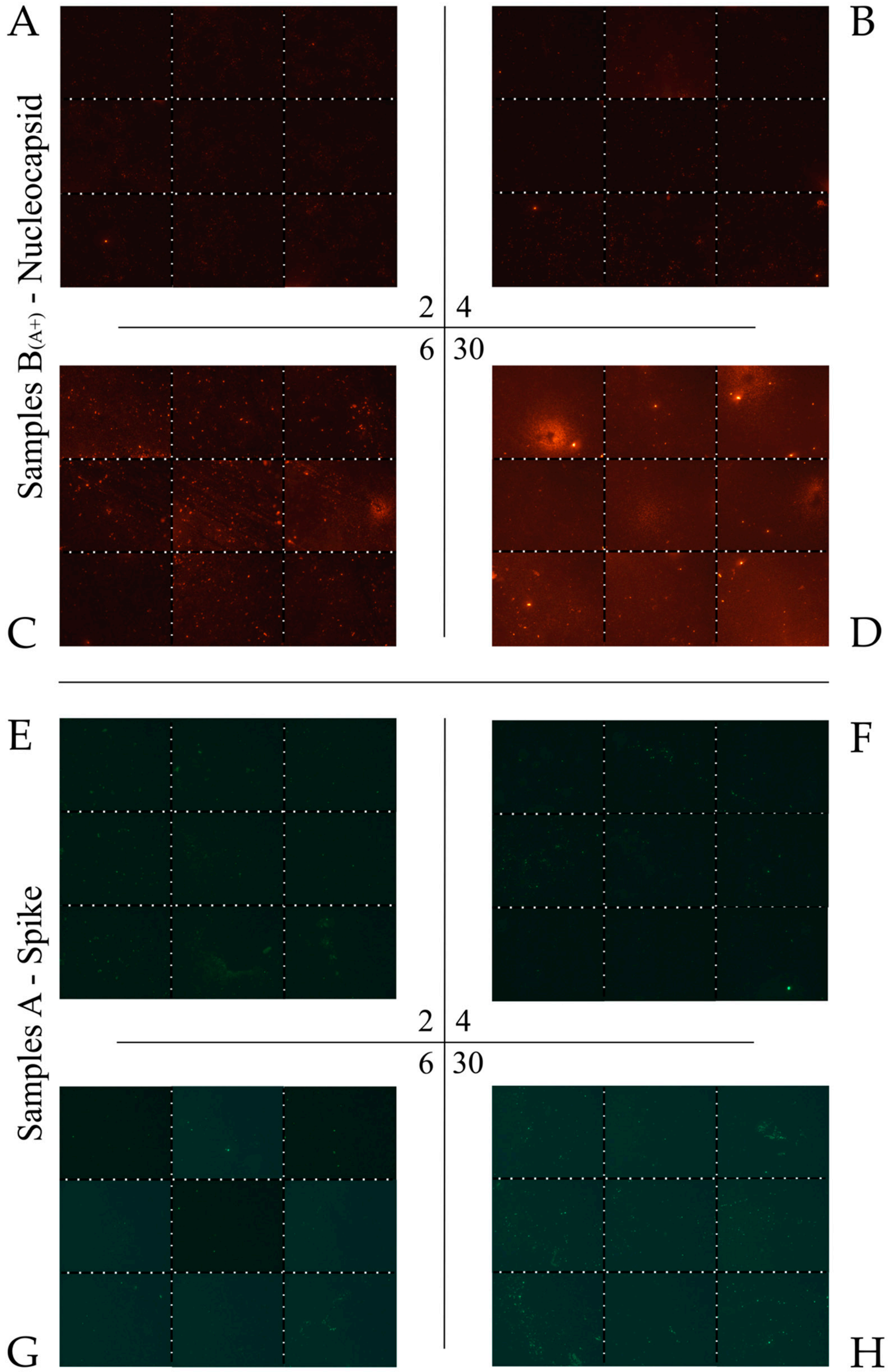

Figure S2 (supplementary materials, upper part) shows the immunofluorescences of sample B $_{(A+)}$ with the nucleocapsid signal increasing from day 2 to day 30 of bacterial culture (Panel A-D). The lower part shows the immunofluorescence of the spike protein - increasing from day 2 to day 30 in samples A of bacterial culture (Panel E-H). **Panel A**: samples B derived from culture after 2. **Panel B**: samples B from cultures after four days. **Panel C**: samples B from cultures after six days. **Panel D**: samples B from cultures after 30 days. **Panel E-H**: samples A, Spike protein signal, from cultures after 30 days.

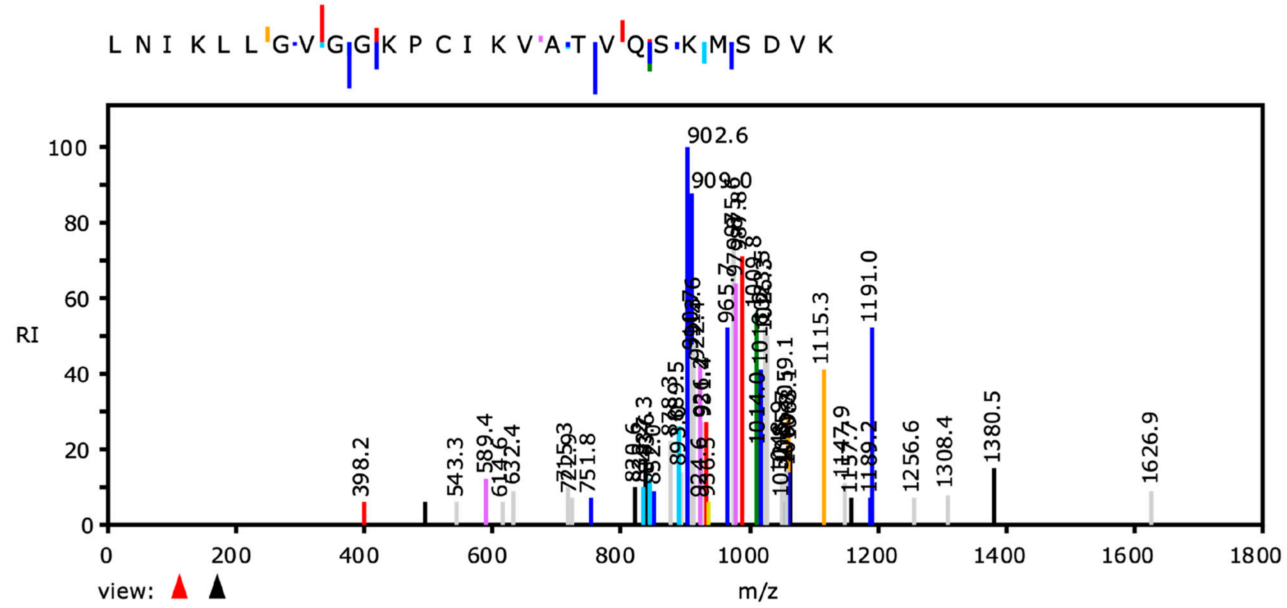

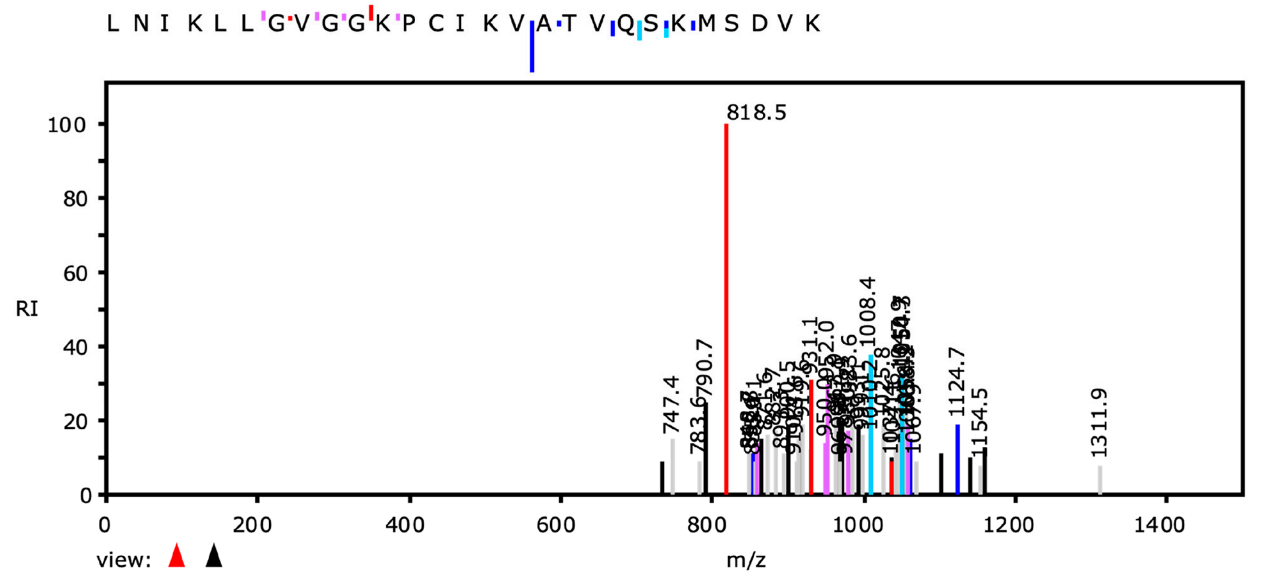

Figure S3: Higher magnification detail of the spectra presented in Figure 4 panel B of the manuscript. The upper image is the sequence: LNIKLLGVGG KPCIKVATVQ SKMSDVK ; the lower image is the sequence: LNIKLLGVGG KPCIKVATVQ SKMSDVK
